# Effect of age and neurofibromatosis type 1 status on white matter integrity in the optic radiations

**DOI:** 10.1093/noajnl/vdaa037

**Published:** 2020-06-25

**Authors:** Peter de Blank, Jeffrey I Berman, Marisa Prelack, John R Sollee, Adam Lane, Amy T Waldman, Michael J Fisher

**Affiliations:** 1 University of Cincinnati Medical Center Department of Pediatrics, Cincinnati, Ohio, USA; 2 Division of Oncology, Cincinnati Children’s Hospital Medical Center, Cincinnati, Ohio, USA; 3 Department of Radiology, The Children’s Hospital of Philadelphia, Philadelphia, Pennsylvania, USA; 4 Division of Neurology, The Children’s Hospital of Philadelphia, Philadelphia, Pennsylvania, USA; 5 Division of Oncology, The Children’s Hospital of Philadelphia, Philadelphia, Pennsylvania, USA

**Keywords:** diffusion tensor imaging, neurofibromatosis type 1, optic pathway glioma, vision, white matter development

## Abstract

**Background:**

Adults with neurofibromatosis type 1 (NF1) have decreased white matter integrity, but differences in children with NF1 have not been described. Defining normal values for diffusion tensor imaging (DTI) measures, especially in the optic radiations, is important to the development of DTI as a potential biomarker of visual acuity in children with optic pathway glioma. This study examines the effect of age and NF1 status on DTI measures in children.

**Methods:**

In this retrospective study, MR imaging including DTI was conducted in 93 children (40 children with NF1 and 53 healthy controls) between 0 and 14 years of age. Regression models of age, sex, and NF1 status on DTI measures were evaluated, and tract-based spatial statistics (TBSS) compared DTI measures in age-matched NF1 to non-NF1 cohorts.

**Results:**

Fractional anisotropy, radial diffusivity, and mean diffusivity in white matter tracts of the optic radiations varied with age and were best modeled by a logarithmic function. Age-related DTI measure change was different in NF1 versus non-NF1 subjects. Normal values and 95% confidence intervals for age 0.5–12 years were derived for both groups. Differences in DTI measures between NF1 and non-NF1 groups at a range of ages were shown diffusely throughout the cerebral white matter using TBSS.

**Conclusions:**

Children with NF1 demonstrate increased diffusion throughout the brain compared to children without NF1 suggesting a potentially altered developmental trajectory of optic radiation microstructure. Defining normal values for white matter integrity in children with NF1 may help target early intervention efforts in this vulnerable group.

**Key Points**

1. Children with NF1 demonstrate differences in white matter integrity across ages.
2. Differences in maturation are found diffusely throughout NF1 white matter.
3. Normal DTI values in the optic radiations (a biomarker of vision) are defined.

**Importance of the Study**

Although adults with neurofibromatosis type 1 (NF1) have been shown to have disordered white matter integrity related to cognitive deficits, the effect of NF1 status on white matter microstructure in young children is poorly understood. Understanding the effects of age and NF1 status on diffusion measures in the optic radiations may be particularly important, as diffusion tensor imaging (DTI) has recently been used as a biomarker of vision in children with NF1-associated optic pathway glioma to help determine indications for treatment. This study describes DTI measures in 93 healthy children (between 0 and 14 years) with and without NF1 throughout the brain with particular attention to the optic radiations. Understanding typical values for white matter integrity in children with NF1 may help target early intervention efforts toward periods of special vulnerability and help refine a noninvasive biomarker to identify children at risk for vision loss due to optic pathway glioma.

Neurofibromatosis type 1 (NF1) is the most common genetic cancer predisposition syndrome, occurring in 1:3000 individuals. Individuals with NF1 often face neurocognitive deficits affecting attention, executive function, and IQ, as well as behavioral abnormalities similar to autistic spectrum disorder.^1–3^ Adults with NF1 exhibit altered white matter integrity as measured by diffusion tensor imaging (DTI)^4^ and are sensitive to insults that may affect white matter.^5^ Previous studies have shown that DTI abnormalities in NF1 correlate with neurocognitive deficits.^6,7^ However, it is unclear whether these differences in white matter integrity are found in developing children or occur later in life. Understanding the trajectory of white matter development in children with NF1 may help target early intervention efforts and identify at-risk children with NF1.

Importantly, diffusion measures in the optic radiations have also been recently used as a biomarker of visual acuity in children with optic pathway glioma (OPG), a low-grade glioma affecting the optic nerve, chiasm, and/or optic tracts and radiations. Approximately 15–20% of children with NF1 will develop an OPG,^8,9^ and the development of quantitative biomarkers of vision in OPG, particularly in young children, is crucial to help understand how to treat and prevent vision loss.^10^ DTI measures in the optic radiations and chiasm in children with OPG have been associated with current visual acuity and future vision loss.^11–13^ However, defining thresholds that would identify abnormal diffusion in an individual patient is challenging because DTI measures change with age and may be affected by NF1 status in young children.^14,15^

This study was conducted to compare the trajectory of white matter maturation in young children with and without NF1 and to describe normal values for DTI measures in the optic radiations. Understanding differences in white matter microstructure and integrity in children with NF1 may help define children at risk for cognitive and behavioral deficits and will help identify children with OPG and abnormal vision.

## Materials and Methods

### Participants

In this study approved by the institutional review board (IRB) of the Children’s Hospital of Philadelphia (CHOP), we retrospectively collected diffusion imaging on 93 individual children between 0 and 14 years of age. NF1 participants without OPG were identified from the clinical registries of the NF, oncology, and neurology clinics. For the healthy control cohort, potential participants were identified through a data capture of electronic records if they had a normal MRI scan (further inclusion and exclusion criteria for this group are described below). Participants were selected based on their age, NF1 status, and presence of evaluable DTI data to ensure even distribution, with up to 5 participants per age group in NF1 and non-NF1 cohorts. Each participant was used only once in the analysis. Because dramatic differences in myelination may be seen especially in early childhood, age groups were divided every 6 months until 3 years, then annually until 14 years.

A chart review was conducted to ensure that potential participants did not have a history of CNS tumors or prematurity less than 35 weeks of gestational age. Participants were also excluded if they had a neurologic disease that might affect white matter integrity (including increased intracranial pressure requiring ventriculoperitoneal shunt, structural brain abnormality, epilepsy, multiple sclerosis, or prior brain surgery/biopsy) or an ophthalmologic disease that might affect vision (including amblyopia, cataract, glaucoma, or retinopathy of prematurity).

Healthy participants with NF1 most commonly underwent brain MRI due to concern for headache or vision loss, in keeping with recommendations for surveillance in NF1 patients.^10,16^ NF1 participants were not excluded for focal areas of signal intensity (FASI) common in NF1. Screening studies of NF1 have shown that OPG are usually apparent on MRI by 15 months of age even if symptoms generally present later in childhood; therefore, occult OPG are unlikely in this cohort.^8^

Healthy participants without NF1 were identified either from a previous series of healthy volunteers (26 subjects) or for a clinical concern (27 subjects). To identify healthy participants without NF1, a data capture was performed using the search terms for “unremarkable” or “normal” and “MRI brain” consistent with reporting language used at the CHOP in patients aged 0–14 years. Non-NF1 patients with central nervous system anatomic or vascular abnormalities, as well as those with microcephaly, systemic or chronic diseases, were also excluded. In non-NF1 patients where the clinical indication for MRI could be identified (missing in 4 patients), the most common reasons for scan included headache (*N* = 7), extracranial mass or injury (*n* = 4), gait abnormality (*n* = 3), and vomiting (*n* = 2). Less common reasons included hypotonia, central apnea, swallow abnormality, inconsolable crying, seizure in the setting of an extracranial infection, and aplasia cutis (*n* = 1 each).

### Diffusion Imaging

DTI was obtained on 4 different Siemens 3T MR scanners: Trio (*n* = 40 scans), Verio (*n* = 41 scans), Skyra (*n* = 12 scans), and Prisma (*n* = 2). The diffusion MR was acquired either as a part of the routine clinical exam performed at the CHOP (*n* = 67) or from de-identified participants as part of an IRB-approved study of healthy children (*n* = 26). Diffusion MR was acquired with an echo-planar pulse sequence with axial 128 × 128 matrix, voxel volume of 6.1–10 mm,^3^ diffusion weighting of *b* = 1000 s/mm^2^, and full brain coverage with no gap between slices. Thirty gradient directions were used in all cases. On the Trio, echo time (TE) was 81–94 ms, with relaxation time (TR) of 7.1–11.6 s and bandwidth of 1395 Hz/pixel. On the Skyra, TE was 70–84 ms, with TR of 7.0–9.4 s and bandwidth of 1565 Hz/pixel. On the Verio, TE was 9–104 ms, with TR of 9.9–14 s and bandwidth of 1395–1445 Hz/pixel. On the Prisma, TE was 55 ms, with TR of 5.4 s and bandwidth of 2300 Hz/pixel.

Postprocessing DTI analysis was performed using a previously described method for automated probabilistic tractography of the optic radiations.^17^ Briefly, using FSL version 5.0.7 (FMRIB), *b* = 0 maps were registered to a standard space (Montreal Neurologic Institute 152 T1 map with 2 mm slice thickness). The inverse of the transformation matrix was then used to apply standard space regions in the lateral geniculate nucleus and occipital pole as well as exclusion regions outside of the pathway of the optic radiations to participants’ *b* = 0 maps. Probabilistic streamline fiber tracking (PROBTRACKX) was used with 1000 starting points seeded in each voxel of the lateral geniculate nucleus, a waypoint in the occipital pole, and exclusion masks. No connectivity threshold was used and tracts were not manually trimmed. Mean fractional anisotropy (FA), radial diffusivity (RD), and mean diffusivity (MD) of the optic radiations were calculated as a weighted average based on the probabilistic density of fibers in each voxel.

Voxelwise statistical analysis of FA data was accomplished using Tract-Based Spatial Statistics (TBSS)^18^ in all participants adjusting for the natural logarithm of age. A nonlinear registration tool was used to align all participants’ FA data to a common space. A mean FA skeleton was created to represent the cores of white matter tracts from the mean FA image. Individual FA data were projected onto this skeleton and voxelwise cross-subject statistical analysis was performed to compare DTI measures (FA, RD, and MD) in NF1 and non-NF1 groups.

### Statistical Analysis

Regression models of age, sex, and NF1 status on DTI measures (FA, RD, and MD) were considered. Multiple models were considered (including linear, exponential, logarithmic, and quadratic models) and the best fit model (determined by *R*^2^) was used. An a priori threshold of *P* < .2 was used to determine the significance of the interaction term of age*NF1 status in the optic radiations. If an interaction was present, the effect of age on DTI measures would be determined separately for NF1 and non-NF1 cohorts. TBSS was used to perform a voxel-by-voxel comparison of NF1 and non-NF1 DTI measures within the skeletons of major white matter tracts. A generalized linear model was created using age, NF1 status, and NF1*ln(age) as covariates to predict FA, RD, and MD. *P*-values for the interaction term NF1*ln(age) were displayed in each voxel in the FA skeleton with a threshold of *P* < .05. Analyses were adjusted for multiple comparisons across space using family-wise error correction and threshold-free cluster enhancement.^18,19^ Derived models from the optic radiations were used to calculate mean values for FA, RD, and MD in the optic radiations at various ages along with associated confidence intervals (CIs). A sensitivity analysis was performed to examine whether areas of T2 hyperintensity common in NF1 had affected the analysis of the optic radiations. In this analysis, NF1 participants who had tracts intersecting areas of hyperintensity seen on *b* = 0 maps were excluded (6 patients excluded, aged 2, 4, 4, 5, 7, and 8 years old). Finally, proposed FA and RD thresholds were applied to a previously reported dataset of DTI measures in 50 children with NF1-OPG to examine test characteristics in a real-world scenario.^11^

## Results

A total of 93 DTI datasets were analyzed, including 40 children with NF1 and 53 children without NF1. Approximately half of all participants were male (21/40 of NF1 participants and 28/53 of non-NF1 participants), and there was no significant difference in sex ratio (*P* = .8, chi-square test) or scanner type (*P* = .7, Fisher’s exact test) between groups. The age distribution of NF1 and non-NF1 groups is provided in Table 1.

**Table 1.** Age and Sex Distribution for Subjects With and Without NF1

Age (Years)	Non-NF1		NF1	
	*n*	*n* (Male)	*n*	*n* (Male)
0–<0.5	3	0	1	1
0.5–<1	0	0	6	4
1–<1.5	3	2	2	0
1.5–<2	1	0	2	0
2–<2.5	3	2	3	2
2.5–<3	0	0	3	2
3–<4	5	2	1	1
4–<5	5	1	2	1
5–<6	7	6	2	2
6–<7	2	0	1	0
7–<8	2	1	5	3
8–<9	1	0	3	0
9–<10	5	2	3	1
10–<11	3	3	2	2
11–<12	5	2	3	0
12–<13	3	1	1	0
13–<14	5	4	0	0

**Figure 1.**
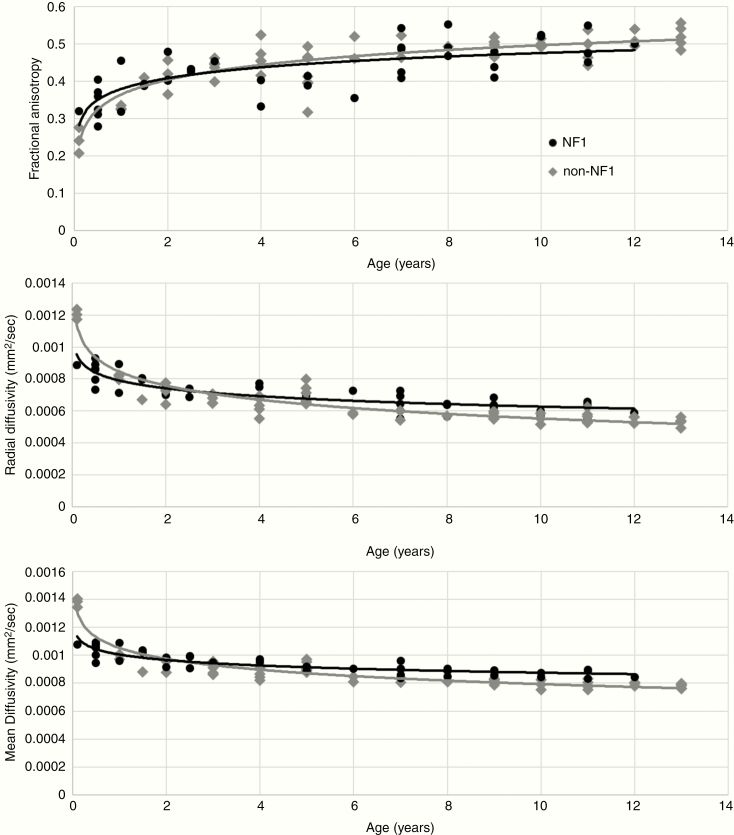
Scatterplots of DTI measures of the optic radiations (FA, MD, and RD) versus age in children with NF1 (black circles) and without NF1 (gray diamonds) including trendlines for NF1 (black) and non-NF1 (gray).

To examine changes to DTI measures in tracts associated with visual acuity in NF1-associated OPG, FA, RD, and MD were measured in the optic radiations for each participant. DTI measures were plotted against participant age (Figure 1). Multiple models for the relationship between age and DTI measures were considered, including linear, exponential, logarithmic, and quadratic. A logarithmic model was chosen as the best fit model as measured by *R*^2^. Multiple regression analysis examining the effect of ln(age), sex, NF1 status, and the interaction term [ln(age)*NF1 status] on FA, MD, and RD was performed. Sex was not a significant covariate, but the interaction term for ln(age)*NF1 status met the a priori threshold for significance of *P* < .2: FA *P* = .16, RD *P* < .001, MD *P* < .001. Therefore, the effect of ln(age) on DTI measures was modeled separately for participants with and without NF1. In a sensitivity analysis, scans in which optic radiation tracts overlapped with areas of signal abnormality seen on *b* = 0 maps were excluded and similar logarithmic relationships between DTI measures and age were found. Regression models of DTI measures versus age, adjusted for sex in children with and without NF1 can be found in Table 2. Although similar logarithmic relationships fit both cohorts, *R*^2^ values were lower for the NF1 compared to the non-NF1 group likely reflecting increased heterogeneity found in this disease.

**Table 2.** Regression Models for DTI Measures of the Optic Radiations (FA, MD, and RD) and Age, Adjusted for Sex in Children With and Without NF1

	Non-NF1 Subjects				NF1 Subjects			
	FA = *B*_1_*ln(age) + *B*_2_				FA = *B*_1_*ln(age) + *B*_2_			
		Coeff.	*P*	95% CI		Coeff.	*P*	95% CI
FA	*B*_1_	0.059	<.001	0.050–0.068	*B*_**1**_	0.042	<.001	0.029–0.055
	*B*_2_	0.373	<.001	0.355–0.391	*B*_2_	0.379	<.001	0.358–0.400
	*R* ^2^ = 0.7822				*R* ^2^ = 0.5236			
	MD = *B*_1_*ln(age) + *B*_2_				MD = *B*_1_*ln(age) + *B*_2_			
		Coeff.	*P*	95% CI		Coeff.	*P*	95% CI
MD	*B*_1_ (10^–4^)	−1.12	<.001	−1.2 to −1.00	*B*_1_ (10^–4^)	−0.56	<.001	−0.67 to −0.5
	*B*_2_ (10^–3^)	1.04	<.001	1.02–1.07	*B*_2_ (10^–3^)	1	<.001	0.99–1.02
	*R* ^2^ = 0.8852				*R* ^2^ = 0.7482			
	RD = *B*_1_*ln(age) + *B*_2_				RD = *B*_1_*ln(age) + *B*_2_			
		Coeff.	*P*	95% CI		Coeff.	*P*	95% CI
RD	*B*_1_ (10^–4^)	−1.28	<.001	−1.41 to −1.15	*B*_1_ (10^–4^)	−0.71	<.001	−0.86 to −0.6
	*B*_2_ (10^–3^)	0.84	<.001	0.81–0.86	*B*_2_ (10^–3^)	0.79	<.001	0.77–0.81
	*R* ^2^ = 0.8857				*R* ^2^ = 0.7210			

**Figure 2.**
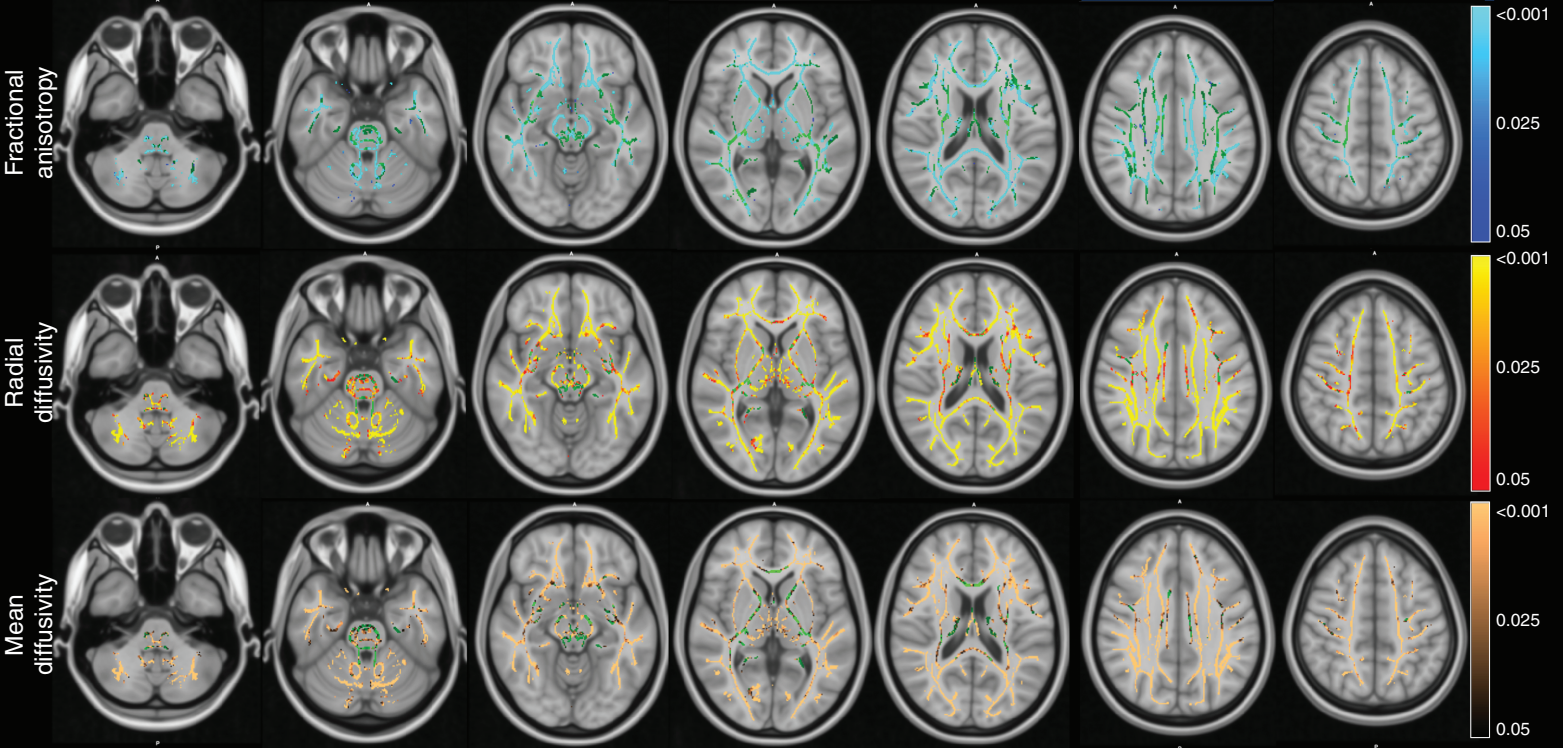
Areas of divergent white matter development (NF1 vs non-NF1) shown on the voxelwise analysis of 93 children. Tracts with significant interaction (*P* < .05) between NF1 status and ln(age) represented by blue (for association with fractional anisotropy), red (radial diffusivity), and copper (mean diffusivity). FA skeleton (green) shows areas without significant differences in developmental trajectory between groups. Images overlaid on the Montreal Neurologic Institute atlas.

To determine whether differences in white matter integrity between NF1 and non-NF1 children were limited to the optic radiations, TBSS was used to compare NF1 and non-NF1 participants, adjusted for ln(age). This analysis identified significant (*P* < .05) reductions in FA and increases in RD and MD among NF1 participants diffusely throughout the white matter skeleton. More importantly, the interaction term NF1*ln(age) for all DTI measures (FA, RD, and MD) was significant in nearly all regions of the white matter skeleton (Figure 2), demonstrating that white matter maturation was affected by NF1 status throughout the brain.

**Figure 3.**
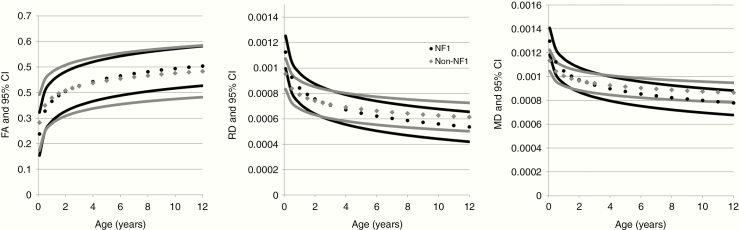
Predicted mean and 95% confidence intervals of DTI measures (FA, MD, and RD) versus age in children with NF1 (black circles) and without NF1 (gray diamonds).

To help determine potential threshold values for abnormal DTI measures in the optic radiations of young children, normal values for age and associated CIs were computed for FA, RD, and MD in the optic radiations among children with and without NF1. Compared to age-matched participants without NF1, NF1 participants demonstrated a different pattern of diffusion measures across young ages, particularly in RD and MD (Figure 3). Although this study does not follow longitudinal development in individual children, these findings may suggest differences in the developmental trajectory of white matter myelination in children with NF1. A table of predicted normal values and 95% CI of DTI measures (FA, RD, and MD) for both NF1 and non-NF1 can be found in Supplementary Table S1.

**Figure 4.**
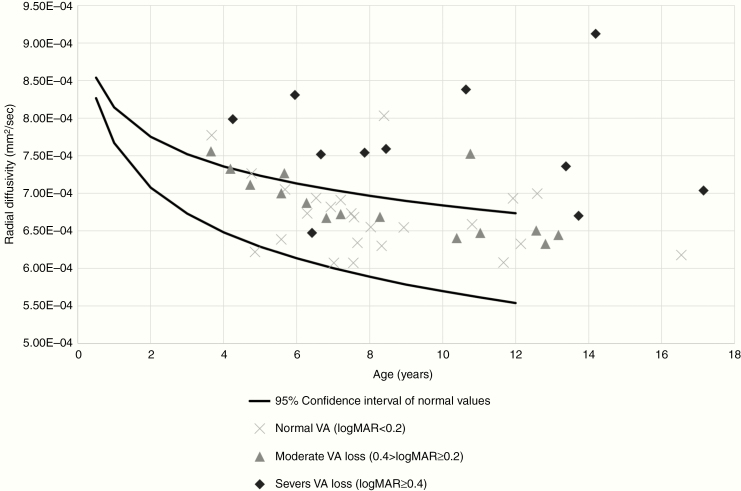
Mean RD of the optic radiations among 50 children with NF1-OPG plotted against the 95% confidence intervals of age-based normal values for NF1 (black lines). Subjects distinguished by visual acuity: severe visual acuity loss (logMAR ≥ 0.4 compared to age-based normal values, black diamond), moderate visual acuity loss (0.4 > logMAR ≥ 0.2 compared to age-based normal values, gray diamonds), and normal visual acuity (logMAR < 0.2 compared to age-based normal values, light gray X).

Finally, to examine the efficacy of an age-based threshold for DTI values, 95% CI of RD for normal NF1 participants was applied to data from a previously published cohort of children with an NF1-associated OPG (Figure 4).^11^ Of 24 patients with normal visual acuity (logMAR < 0.2 compared to age-based norms), 20 (83.3%) had mean RD of the optic radiations below the upper 95% CI for age. Among 11 patients with severe visual acuity loss (logMAR ≥ 0.4), 10 (91%) had RD above the 95% CI for age. Among 15 patients with moderate visual acuity loss (0.2 ≥ logMAR > 0.4), findings were intermediate with 3 (20%) participants with RD above the 95% CI for age.

## Discussion

Our results reveal varied patterns of FA, RD, and MD measures with advancing age between healthy children with and without NF1. While these differences were less notable for FA (*P* = .16, less than our a priori threshold), the effect of NF1 status made a significant effect (*P* < .001) on RD and MD measures. The significant interaction term NF1*ln(age) in this cross-sectional study suggests that white matter tracts in young children with NF1 may follow a different developmental trajectory than those without NF1. Within the optic radiations, diffusion measures in infants appeared reduced in NF1 participants (reflected in the *B*_2_ coefficient in Table 2) but matured more slowly than participants without NF1 (reflected in the *B*_1_ coefficient). Although this study does not follow longitudinal development in individual children, these findings suggest that children with NF1 may have an altered and delayed maturation of white matter in the developing brain, and differences in white matter myelination and heterogeneity lead to important differences in thresholds for normal DTI measures in NF1 and non-NF1 participants.

Adults with NF1 have previously been shown to have abnormal white matter microstructure as measured with DTI. Among 10 adult participants with NF1 and 10 healthy adult controls, NF1 was associated with reduced FA values throughout multiple individual regions of interest.^20^ This finding has been recently corroborated in 14 adults with NF1 and 12 unaffected controls which demonstrated that decreased white matter integrity associated with NF1 can be found diffusely throughout the majority of white matter tracts.^4^ However, these investigations have been limited to adult participants and have been unable to assess differences in DTI measures in the developing brain. Similarly, studies of children have examined smaller patient samples and have not examined the effect of age and NF1 on diffusion measures in children.^21^ The current study advances our understanding of the dynamic and abnormal maturational processes which result in pervasive white matter abnormalities in adults with NF1. In our examination of the optic radiations, we find children with NF1 appear to develop increased diffusion (as seen in adults) by school age (>5 years of age). This suggests that differences found in NF1 white matter microstructure may not be static throughout a child’s life; however, further longitudinal studies are necessary to determine whether a specific window of opportunity during childhood exists for targeted treatment to delay or moderate these differences.

Importantly, differences in NF1 DTI measures were not due to abnormalities obvious on T2-weighted imaging. Areas of T2 hyperintensity, also called FASI, are commonly seen in children with NF1 in the cerebellum, basal ganglia, and brainstem and frequently disappear in adulthood. The pathology of these lesions has revealed increased vacuolization that may influence diffusion measures.^22^ Previous studies examining the effect of T2 hyperintensities on diffusion measures have demonstrated mixed results, suggesting that FASI may affect DTI measures in some but not all brain regions.^23–25^ Because FASI may be associated with interruptions in tractography,^26^ a sensitivity analysis was performed eliminating participants whose optic radiations intersect with FASI. Importantly, excluding these participants did not eliminate differences between NF1 and non-NF1 participants, suggesting that differences are not caused exclusively by lesions obvious on T2-weighted images. However, microscopic changes in vacuolization not evident on T2-weighted imaging may explain differences in NF1 diffusion measures compared to non-NF1, as well as the increased variability of these measures in children with NF1. We have attempted to reduce this variability in young NF1 participants by categorizing age differences every 6 months rather than every year.

The definition of age-based normal values for DTI measures is a fundamental step in developing DTI biomarkers in NF1. Noninvasive biomarkers of cognition or vision in children with NF1 could help identify children at need for early intervention but must be contextualized with age-based normal values. DTI measures in children with NF1 have recently been associated with aspects of processing speed and inhibitory control.^5–7^ In this study, we focused on DTI measures in the optic radiations as a biomarker of visual acuity in children with NF1-associated OPG. Previous studies have demonstrated a strong association between DTI of the optic radiations and visual acuity in children with OPG when adjusted for age^11,12^; however, the clinical utility of this association is limited in individual cases without age-based normal values. We applied ranges derived in this study to a previously published cohort to show that 10 of 11 children with severe vision loss (logMAR ≥ 0.4 from age-based norms) had RD greater than the 95% CI for age. These thresholds may become clinically useful for individual studies that follow a similar DTI acquisition and semi-automated postprocessing.^17^

This study is subject to important limitations. The cohort recruited for this study was scanned on multiple MR scanners. Although DTI measures are sensitive to acquisition parameters, the ratio of scanner types used in each group was not significantly different (*P* < .7, Fisher’s exact test). Thus, although the use of multiple scanner types may increase variance, it is not expected to introduce a group-level bias. While this cohort represents one of the largest DTI evaluations of normal children with and without NF1, some age groups remain underrepresented. Normal diffusion measures presented in this study are derived from mathematical models, and age groups with the least representations may have the least stable predicted normal values. For this reason, normal values in the youngest and oldest age groups (<0.5 and >12 years) were not reported as these may be subject to high variance due to the small sample size. Further studies could help refine the differences between groups, especially in early age groups. Measures of white matter integrity may be associated with unmeasured cognitive differences between participants. We have attempted to limit this affect by focusing on the optic radiations that are important to vision but have a limited effect on cognition. This cohort also represents a convenience sample of children undergoing brain MRI. While chart review was conducted to exclude conditions that may affect DTI measures, some participants may still have abnormal DTI due to occult pathology. Ages were categorized by 6-month or 12-month intervals to preserve confidentiality; however, more definition in age may have led to reduced variability in DTI measures in each age group. Although this study demonstrates that white matter integrity changes with age and is different in children with NF1, the specific DTI threshold values will be most usefully applied to studies that replicate the DTI acquisition parameters and postprocessing techniques used in this study.^17^ These parameters are used to prospectively study visual acuity loss in OPG, and other biomarkers in other brain regions will need to be defined separately.

Measures of diffusion vary with age and development. Children with NF1 demonstrate differences in DTI values (FA, RD, and MD) with age compared to children without NF1, suggesting potential differences in the developmental trajectory of white matter microstructure due to NF1. Differences in DTI measures can be seen across the ages studied and in the youngest children. Defining normal values for white matter development may help identify children at risk for neurocognitive or visual deficits. DTI biomarkers in NF1 require further prospective validation but offer the potential for earlier intervention in this vulnerable population.

## Supplementary Material

vdaa037_suppl_Supplementary_Table_1
