# Supplementary material for: Effect of age and neurofibromatosis type 1 status on white matter integrity in the optic radiations
Source: Neurooncol Adv. 2020 Jun 25;2(Suppl 1):i150–8. doi: 10.1093/noajnl/vdaa037 (PMC7317057; doi:10.1093/noajnl/vdaa037)
Supplement: vdaa037_suppl_Supplementary_Table_1 [file vdaa037_suppl_supplementary_table_1.pdf]

Supplemental Table 1. Mean DTI measures (FA, RD, MD) and 95% confidence intervals for subjects with and without NF1 between 0.5 -12 years of age

Non-NF1

Age (yrs)	Mean FA	95%CI	Mean RD (x10^{-4} mm^2/sec)	95%CI	Mean MD (x10^{-4} mm^2/sec)	95%CI
0.5	0.327	0.248-0.407	9.27	8.07-10.5	11.2	10.2-12.3
1	0.366	0.287-0.444	8.42	7.23-9.61	10.5	9.44-11.5
1.5	0.388	0.311-0.466	7.92	6.74-9.10	10.0	9.00-11.1
2	0.404	0.327-0.482	7.57	6.39-8.75	9.73	8.70-10.8
2.5	0.467	0.339-0.494	7.30	6.12-8.47	9.49	8.46-10.5
3	0.427	0.350-0.504	7.07	5.90-8.24	9.29	8.26-10.3
4	0.443	0.366-0.520	6.72	5.55-7.89	8.98	7.95-10.0
5	0.455	0.378-0.533	6.44	5.27-7.62	8.74	7.71-9.77
6	0.466	0.388-0.543	6.22	5.05-7.39	8.54	7.52-9.57
7	0.474	0.397-0.551	6.03	4.86-7.20	8.38	7.35-9.40
8	0.482	0.404-0.559	5.87	4.69-7.04	8.23	7.21-9.26
9	0.488	0.411-0.565	5.72	4.55-6.90	8.11	7.08-9.13
10	0.494	0.416-0.571	5.59	4.42-6.77	7.99	6.96-9.02
11	0.499	0.422-0.577	5.48	4.30-6.65	7.89	6.86-8.92
12	0.504	0.426-0.582	5.37	4.19-6.55	7.80	6.76-8.83

NF1

Age (yrs)	Mean FA	95%CI	Mean RD (x10^{-4} mm^2/sec)	95%CI	Mean MD (x10^{-4} mm^2/sec)	95%CI
0.5	0.3498	0.247-0.452	8.40	7.27-9.53	10.4	9.60-11.3
1	0.379	0.278-0.480	7.91	6.79-9.02	10.0	9.23-10.9
1.5	0.396	0.296-0.496	7.62	6.51-8.72	9.82	9.00-10.6
2	0.408	0.308-0.508	7.41	6.31-8.51	9.66	8.84-10.5
2.5	0.417	0.318-0.517	7.26	6.15-8.36	9.53	8.72-10.3
3	0.425	0.325-0.525	7.13	6.03-8.23	9.43	8.61-10.2
4	0.437	0.337-0.537	6.92	5.82-8.02	9.27	8.45-10.1
5	0.446	0.347-0.546	6.76	5.66-7.86	9.14	8.33-9.95
6	0.454	0.354-0.554	6.63	5.53-7.74	9.04	8.22-9.85
7	0.461	0.360-0.561	6.52	5.42-7.63	8.95	8.13-9.77
8	0.466	0.366-0.567	6.43	5.32-7.54	8.88	8.06-9.69
9	0.471	0.371-0.572	6.34	5.23-7.46	8.81	7.99-9.63
10	0.476	0.375-0.576	6.27	5.16-7.38	8.75	7.93-9.57
11	0.480	0.379-0.581	6.20	5.09-7.31	8.70	7.87-9.52
12	0.483	0.382-0.584	6.14	5.02-7.26	8.65	7.82-9.47
